# Osteochondral Regeneration with 3D‐Printed Biodegradable High‐Strength Supramolecular Polymer Reinforced‐Gelatin Hydrogel Scaffolds

**DOI:** 10.1002/advs.201900867

**Published:** 2019-06-11

**Authors:** Fei Gao, Ziyang Xu, Qingfei Liang, Haofei Li, Liuqi Peng, Mingming Wu, Xiaoli Zhao, Xu Cui, Changshun Ruan, Wenguang Liu

**Affiliations:** ^1^ School of Materials Science and Engineering Tianjin Key Laboratory of Composite and Functional Materials Tianjin University Tianjin 300350 China; ^2^ Research Center for Human Tissue and Organs Degeneration Institute Biomedical and Biotechnology Shenzhen Institutes of Advanced Technology Chinese Academy of Sciences Shenzhen 518055 China

**Keywords:** 3D printing, biohybrid gradient scaffolds, high strength, osteochondral regeneration, supramolecular polymers

## Abstract

Biomacromolecules with poor mechanical properties cannot satisfy the stringent requirement for load‐bearing as bioscaffolds. Herein, a biodegradable high‐strength supramolecular polymer strengthened hydrogel composed of cleavable poly(*N*‐acryloyl 2‐glycine) (PACG) and methacrylated gelatin (GelMA) (PACG‐GelMA) is successfully constructed by photo‐initiated polymerization. Introducing hydrogen bond‐strengthened PACG contributes to a significant increase in the mechanical strengths of gelatin hydrogel with a high tensile strength (up to 1.1 MPa), outstanding compressive strength (up to 12.4 MPa), large Young's modulus (up to 320 kPa), and high compression modulus (up to 837 kPa). In turn, the GelMA chemical crosslinking could stabilize the temporary PACG network, showing tunable biodegradability by adjusting ACG/GelMA ratios. Further, a biohybrid gradient scaffold consisting of top layer of PACG‐GelMA hydrogel‐Mn^2+^ and bottom layer of PACG‐GelMA hydrogel‐bioactive glass is fabricated for repair of osteochondral defects by a 3D printing technique. In vitro biological experiments demonstrate that the biohybrid gradient hydrogel scaffold not only supports cell attachment and spreading but also enhances gene expression of chondrogenic‐related and osteogenic‐related differentiation of human bone marrow stem cells. Around 12 weeks after in vivo implantation, the biohybrid gradient hydrogel scaffold significantly facilitates concurrent regeneration of cartilage and subchondral bone in a rat model.

## 1 Introduction

Articular cartilage defects along with subchondral bone degeneration in the knee joint is a common clinical problem, which leads to knee joint dysfunction, significant pain, and even disability.^1^, ^2^, ^3^, ^4^ What is worse, the degeneration of subchondral bone in cartilage defect may further aggravate osteoarthritis.^5^, ^6^, ^7^, ^8^ Thus, the success of osteochondral regeneration is largely dependent upon constructing a scaffold with an ability to recapitulate niche cues. Hydrogels are emerging as a promising class of biomaterials for both soft and hard tissue regeneration.^9^, ^10^, ^11^, ^12^, ^13^ Recently, with rapid development of 3D printing technology, increasing studies, including ours, are focused on fabricating scaffolds by 3D printing of hydrogel bioinks for osteochondral repair.^14^, ^15^, ^16^, ^17^ Among a wide array of hydrogels for tissue engineering, gelatin hydrogel has been developed as a variety of bioinks for 3D printing due to its better biocompatibility, biodegradability, bioactivity, and abundance from diverse sources. However, physical gelatin hydrogel itself or extensively used chemically crosslinked methacrylated gelatin (GelMA) hydrogels are weak and brittle in mechanical properties, precluding their applications as load‐bearing scaffolds.^18^, ^19^, ^20^ Recently, mechanically resilient gelatin hydrogels crosslinked by supramolecular host–guest interactions^21^ or dual synergistic physical crosslinking^22^ were reported by Bian. GelMA/β‐tricalcium phosphate composite hydrogel was also developed and explored as a bone graft material.^23^ However, very few studies were devoted to fabricating high strength gelatin hydrogels and thus developing printable bioinks for osteochondral reconstruction.

To treat osteochondral defect, our team constructed a biohybrid scaffold by one‐step thermal‐assisted extrusion printing of supramolecular poly(*N*‐acryloyl glycinamide)‐based hydrogel bioink mixed with organic nanoparticles.^24^, ^25^ However, to make the hydrogel printable, the hydrogen bonding interactions were unavoidably sacrificed, thus resulting in a marked decrease in the mechanical strength of printed constructs far below that of native cartilage. Moreover, this hydrogel was not degraded due to the stable hydrogen bonding interactions, and the printed bare scaffold did not support cell attachment either; thus, it exists as the foreign matter in the defect without integrating with the surrounding tissues. This will severely restrict their applications for osteochondral regeneration. Therefore, developing biodegradable high‐strength hydrogel scaffold for guiding the ingrowth of bone and cartilage tissue to achieve true osteochondral regeneration is highly desired.

**Scheme 1:**
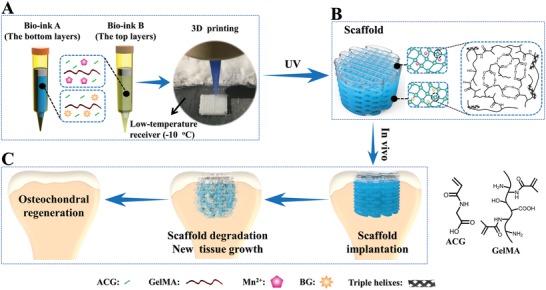
Schematic illustration of 3D printing of the biohybrid gradient scaffolds for repair of osteochondral defect. A) The compositions of bioink A and bioink B, and 3D‐bioprinting method of the biohybrid gradient scaffolds assisted with a low‐temperature receiver; B) formation of stable hydrogel scaffold after UV light‐initiated polymerization and main hydrogen bonding interactions in the PACG‐GelMA network; C) the repair of osteochondral defects treated with the biohybrid gradient PACG‐GelMA hydrogel scaffold with Mn^2+^ and BG being respectively loaded on the top layers and bottom layers in animal experiment.

Motivated to address the above challenges, we aim to design and synthesize a novel biodegradable high strength supramolecular hydrogen bonding strengthened‐gelatin hydrogel, and thus fabricate a gradient hydrogel bioscaffold that is able to provide a mechanical support in the early stage of osteochondral repair, and eventually degraded along with the ingrowth of the new tissue. Previously, inspired by remarkable stimuli‐responsive changes in consistency of sea cucumbers, we prepared supramolecular poly (*N*‐acryloyl 2‐glycine) (PACG) hydrogel with side chain containing amide and carboxyl dual hydrogen bonds, which not only contributed to strengthening the hydrogels formed, but also served as dynamic bonds to modulate the mechanical properties of the hydrogels.^26^ Nevertheless, at a physiological neutral pH, the PACG hydrogel was quickly autolyzed and disintegrated within several hours, making it unsuitable for tissue repair scaffold application. In light of strengthening mechanism and dynamic cleavability of PACG, we propose to develop a novel supramolecular hydrogen bonding strengthened GelMA chemical crosslinked hydrogel (PACG‐GelMA) by incorporating the reversible hydrogen bonds of ACG into the GelMA hydrogel system. The dual hydrogen bonds of PACG side chain can reinforce and stabilize the GelMA network, and in turn the chemical crosslinking of GelMA prolongs the degradation of PACG network. It is anticipated that the PACG‐GelMA hydrogel will be eventually degraded in vivo due to the final degradability of stabilizing GelMA. Furthermore, owing to the thermoreversible gel⇔sol transition behavior and tunable viscosity suited for 3D printing, gelatin mixed with ACG monomer is used as a bioink. In this work, a series of biodegradable preprogrammed biohybrid gradient PACG‐GelMA hydrogel scaffolds are printed, and stabilized with UV light irradiation. To enhance the repair efficacy of cartilage defect, the bioactive manganese ions (Mn^2+^) are loaded into the top cartilage layer of gradient PACG‐GelMA hydrogel scaffolds, while the bioactive glass (BG) is incorporated into the bottom subchondral bone layer of the scaffold with an aim to facilitate new bone regeneration (**Scheme** 1). The mechanical properties of the hydrogels and printed scaffolds are determined. We will focus on evaluating the bioactivity and osteochondral regeneration efficacy of the PACG‐GelMA hydrogel scaffolds in vivo.

## 2 Results and Discussion

### 2.1 Preparation and Characterization of PACG‐GelMA Hydrogels

Prior to printing biohybrid gradient scaffold for treatment of osteochondral defect, we first prepared a supramolecular polymer strengthened chemical crosslinking hydrogel ink by copolymerizing ACG with GelMA. To improve its 3D printability, we synthesized and selected GelMA with low degree of methacrylation (29%, determined by integration of ^1^H NMR spectra, Figure S1, Supporting Information) due to its similar viscosity to that of the pristine gelatin (to be determined in the later rheological measurement). The Fourier‐transform infrared spectroscopy (FTIR) spectra suggested the successful formation of GelMA and PACG‐GelMA hydrogels (Figure S2A, B, Supporting Information).

The problem for in vivo implantation of PACG hydrogel is its quick dissolution at a neutral pH due to the dissociation of carboxyl groups.^26^ In this study, copolymerization of GelMA can maintain the swelling stability of the PACG‐GelMA hydrogel in aqueous solution because of chemical crosslinking networks formed. Figure S3 in the Supporting Information displays the swelling ratios of the hydrogels as a function of time. In general, with an increase of ACG content at fixed GelMA concentration or with an increase of GelMA content at fixed ACG concentration, the swelling ratio and the time to reach a swelling equilibrium of the PACG‐GelMA hydrogels decrease by a different extent. An interpretation is that increasing the contents of ACG and GelMA introduces more H‐bonds of PACG and chemical crosslinks between the PACG and GelMA chains, thus restricting the diffusion of water molecules into the network. Notably, the equilibrium swelling ratio of PACG10‐GelMA10 hydrogels (2.49) is close to that of PACG35‐GelMA7 hydrogels (2.24) possibly due to the similar crosslinking density. Importantly, after swelling equilibrium, the volume of the PACG‐GelMA hydrogels remains very stable without occurrence of further swelling, indicating excellent “nonswellable” properties of the PACG‐GelMA hydrogels, which are critical as an implantable tissue engineering scaffold for early stage of load bearing. The equilibrium water contents (EWCs) of the PACG‐GelMA hydrogels vary from 74% to 98% over the selected range of the monomer concentrations depending on ACG/GelMA ratio (Figure S4, Supporting Information).

**Figure 1:**
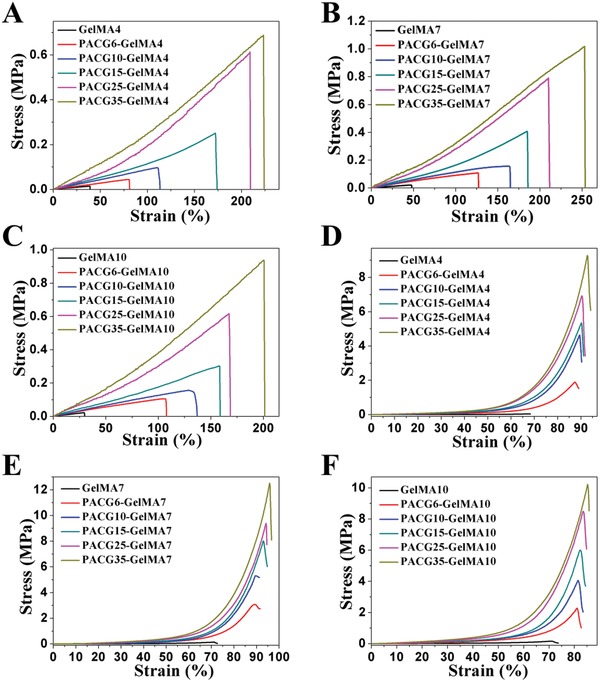
A–C) Tensile stress–strain curves and D–F) compressive stress–strain curves of the PACG‐GelMA hydrogels with varied initial concentrations of ACG and GelMA in deionized water.

The PACG‐GelMA hydrogels happen to be noncovalently strengthened chemically crosslinked hydrogels that combines primarily the covalent GelMA‐PACG crosslinking with PACG hydrogen bonded crosslinking, though there exist PACG‐GelMA hydrogen bonding interactions.^27^, ^28^ **Figure** 1 summarizes the mechanical properties of the fully swollen PACG‐GelMA hydrogels. It can be clearly seen that the mechanical properties of PACG‐GelMA hydrogels are significantly enhanced compared to those of pristine GelMA hydrogels. And when the GelMA content is fixed, the mechanical strengths are considerably increased with the increment in monomer content of ACG. While at a constant ACG content, the mechanical strengths exhibit first an increasing trend and then decrease with increasing GelMA content. Over a 4–7% GelMA range, the mechanical properties of the PACGX‐GelMA7 are better than those of PACGX‐GelMA4; when GelMA content continues to increase from 7% to 10%, the Young's modulus and compressive modulus are further increased, but the tensile strength, break strain, compressive strength, and compressive failure strain decrease slightly. In the selected range of initial concentrations of ACG, the PACGX‐GelMA4 hydrogels achieve 0.018–0.721 MPa tensile strength, 42–224% break strain, 38–252 kPa Young's modulus, 0.048–8.9 MPa compressive strength, 7–471 kPa compressive modulus, and 68–93% compressive failure strain. The PACGX‐GelMA7 hydrogels achieve 0.029–1.108 MPa tensile strength, 52–245% break strain, 49–281 kPa Young's modulus, 0.11–12.4 MPa compressive strength, 27–651 kPa compressive modulus, and 70–97% compressive failure strain. The PACGX‐GelMA10 hydrogels achieve 0.025–0.978 MPa tensile strength, 30–207% break strain, 74–320 kPa Young's modulus, 0.125–9.9 MPa compressive strength, 51–837 kPa compressive modulus, and 72–88% compressive failure strain. It has been proved that there is only covalent crosslinking in the pristine GelMA hydrogel network whose triple helix hydrogen bonds are too weak to act as a strengthening mechanism.^29^, ^30^ Therefore, the pristine GelMA hydrogels are poor in mechanical properties. In comparison, for the PACG‐GelMA hydrogels, the higher mechanical strengths are primarily attributed to multiple hydrogen bonding reinforced mechanism from PACG.^26^, ^31^ However, excessively high crosslinking density of GelMA may interfere with the formation of PACG hydrogen bonds, resulting in a decline of mechanical strength. It is noted that the PACG10‐GelMA10 and PACG35‐GelMA7 hydrogels demonstrate excellent mechanical properties with robust tensile strength (up to 0.158 and 1.1 MPa), large stretchability (up to 139% and 245%), high Young's modulus (up to 143 and 281 kPa), high compressive strength (up to 4.1 and 12.4 MPa), and compressive modulus (up to 421 and 651 kPa). We note that the compressive modulus of PACG10‐GelMA10 hydrogel is in the range of native cartilage's modulus (300–800 kPa).^20^, ^32^, ^33^

**Figure 2:**
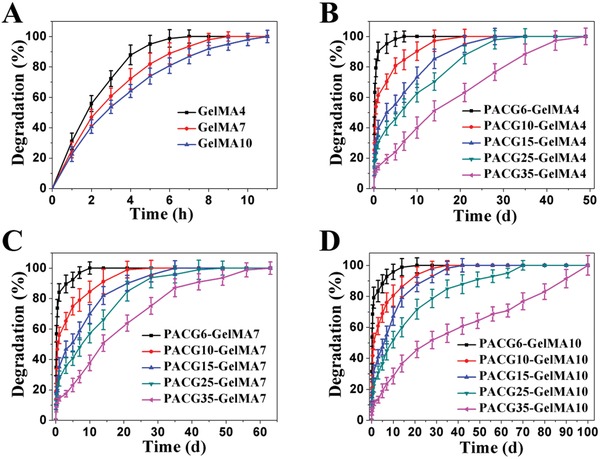
In vitro degradation behaviors of PACGX‐GelMAY hydrogels with varied initial concentrations of ACG and GelMA in collagenase solution.

The PACG‐GelMA hydrogels were shown to be sufficiently stiff and stable in an aqueous medium. Next, to imitate in vivo environment, we evaluated the degradation behaviors of PACG‐GelMA hydrogels in collagenase solution by monitoring the percentage of residual hydrogel mass as a function of time. As shown in **Figure** 2, the GelMA hydrogel is degraded rapidly, and is completely dissolved in solution within 11 h. In contrast, the PACG‐GelMA hydrogels show a slower degradation rate under the same condition. The degradation time varies from 7 to 100 days over the selected range of the initial contents. It is noted that with an increment of ACG at a fixed GelMA content, the degradation rate is decreased significantly. Similarly, raising GelMA feeding at a fixed ACG content also results in a lower degradation rate. It is not difficult to understand that copolymerization of ACG can protect cleavage sites on gelatin backbone from collagenase attack within the copolymer networks. In addition, the enhanced crosslinking density significantly limits the penetration of collagenases into the network, thus slowing down the degradation process.^34^ It is noteworthy that the complete degradation times of PACG10‐GelMA10 and PACG35‐GelMA7 hydrogel are 35 and 63 days, respectively. This suggests that the PACG35‐GelMA7 hydrogel scaffold can provide a longer time of mechanical support before the defected tissue is repaired, meanwhile avoiding the collapse of the scaffold.

### 2.2 Preparation and Characterization of PACG‐GelMA‐Mn^2+^ and PACG‐GelMA‐BG Hybrid Hydrogels

As aforementioned, the PACG10‐GelMA10 and PACG35‐GelMA7 hydrogels exhibit a similar swelling behavior, which can avoid the occurrence of delamination of the printed multilayer scaffolds upon contacting body liquid. In addition, these two hydrogels are sufficiently stiff and degraded within appropriate time. Considering their different degradation time, the PACG10‐GelMA10 hydrogel with a shorter degradation time was used as an ink for printing top cartilage layer, and PACG35‐GelMA7 hydrogel with a longer degradation time served as an ink for bottom bone layer.

Taking into account the bioactivity of Mn^2+^ ions in promoting cartilage synthesis,^35^ Mn^2+^‐doped PACG10‐GelMA10 hydrogel (termed as PACG10‐GelMA10‐Mn^2+^) was prepared. We measured the mechanical properties of the PACG10‐GelMA10‐Mn^2+^ hydrogels, and found no difference with those of PACG10‐GelMA10 hydrogel since only trace amount of ions (0.125–4 ppm) was added, causing no influence on the mechanical strength.

Bioactive glass (BG) has proven to be an important biocompatible bone‐repair material since its released bioactive ions can promote cell adhesion, proliferation, and differentiation.^36^, ^37^ To facilitate bone regeneration, we then prepared bone layer ink by adding bioactive glass into the PACG35‐GelMA7 hydrogel. The FTIR spectra of BG and PACG35‐GelMA7‐BG hybrid hydrogels suggest the successful doping of BG into the hydrogel system (Figure S2C,D, Supporting Information).

Figure S5 in the Supporting Information exhibits the mechanical properties of fully swollen PACG35‐GelMA7‐BG hydrogels with varied BG contents. It is clearly observed that the mechanical strengths decrease with the increment of BG content. It is rational to think that incorporation of BG does not affect the chemical crosslinking, but may interfere with the formation of hydrogen bonds of PACG. Nevertheless, at 1% BG content, 0.71 MPa tensile strength, 7.51 MPa compressive strength, 267 kPa Young's modulus, and 579 kPa compressive modulus can be maintained. Thus, in the subsequent experiments, we chose PACG35‐GelMA7 hydrogel doped with 1% BG (abbreviated as PACG35‐GelMA7‐BG without otherwise statement) as a bone layer ink. We found that only 1% BG exerted a negligible effect on degradation of the hybrid hydrogels.

Cell affinity is a prerequisite for tissue regeneration. Currently reported high‐strength hydrogels rarely support cell adhesion, spreading, and proliferation.^38^, ^39^ Herein, we examined the effects of different concentrations of Mn^2+^ on the proliferation of hBMSCs on the surface of PACG10‐GelMA10 hydrogel. Figure S6A in the Supporting Information shows that there is no statistically significant difference for the cell proliferation over the concentration range of 0–1 ppm, whereas the cell proliferation ability in the group of 1 ppm is slightly lower than that in the 0.5 ppm group. Comparatively, the cell proliferation on the Mn^2+^‐doped hydrogels is significantly different from that on the pure PACG10‐GelMA10 hydrogel at 2 and 4 ppm concentrations of Mn^2+^. Based on the above analysis, we chose PACG10‐GelMA10 hydrogel doped with 0.5 ppm Mn^2+^ (defined as PACG10‐GelMA10‐Mn^2+^ for description simplicity) as the cartilage layer ink. Similarly, PACG35‐GelMA7 and PACG35‐GelMA7‐BG hydrogels can also support the cell proliferation very well, with no significant difference between these two groups (Figure S6B, Supporting Information). From the results of live/dead assay (Figure S6C–F, Supporting Information), we can see that the hBMSCs grow well and show spindle‐like morphologies on the surfaces of the PACG10‐GelMA10, PACG10‐GelMA10‐Mn^2+^, PACG35‐GelMA7, and PACG35‐GelMA7‐BG hydrogels. The above results indicate that introducing GelMA can promote cell adhesion and proliferation on the gel surface.

The rheological characteristics of the PACG10‐GelMA10‐Mn^2+^ (cartilage layer ink) and PACG35‐GelMA7‐BG (bone layer ink) hydrogels were further examined. Figure S7A,B in the Supporting Information shows the frequency scans of PACG10‐GelMA10‐Mn^2+^ and PACG35‐GelMA7‐BG hydrogels. As the frequency increases from 0.1 to 20 Hz, the storage modulus G′ and the loss modulus G″ of both the PACG10‐GelMA10‐Mn^2+^ and PACG35‐GelMA7‐BG hydrogels remain almost constant, and the G′ is far above the G″ (Figure S7A,B, Supporting Information), suggesting gelling properties.^40^, ^41^ In addition, the G′ values of PACG35‐GelMA7‐BG hydrogels are much larger than those of PACG10‐GelMA10‐Mn^2+^ hydrogels, which is consistent with the results of mechanical test above. Figure S7C,D in the Supporting Information shows the recovery ability of PACG10‐GelMA10‐Mn^2+^ and PACG35‐GelMA7‐BG hydrogels under continuous alternate oscillatory shear strain measurements. Under the small shear strain (10%), the G′ of the gel is larger than G″, showing a typical elastic solid characterisitic. When the large shear strain (100%, 300%) is applied, the PACG‐GelMA hydrogel switches into a sol state with the G″ being greater than G′, implying the breakup of the network. Interestingly, the G′ and G″ of the gel can rapidly recover to the initial values after the large shear strain is removed, indicating the strong chemical crosslinks and reversible multiple hydrogen bonding interactions contribute to the excellent recovery ability of the network.^42^, ^43^ This quick recovery capability owned by PACG10‐GelMA10‐Mn^2+^ and PACG35‐GelMA7‐BG make them ideal inks for printing gradient scaffold for treating osteochondral defect.

### 2.3 3D Printing of Biohybrid Hydrogel Scaffolds

**Figure 3:**
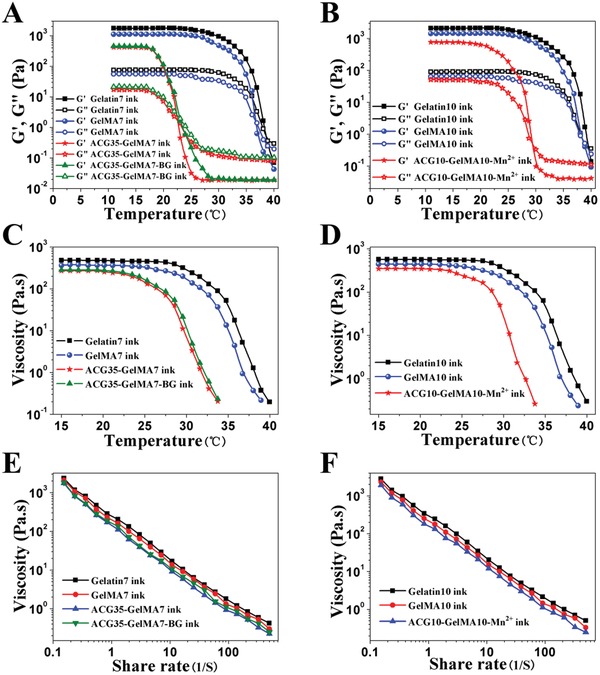
A,B) Influences of the concentration of GelMA and ACG, and addition of BG on the rheological properties of the hydrogels. Variation in dynamic storage moduli G′ and loss moduli G″ as a function of temperature in a temperature amplitude sweep test, where the cross‐over points between the gel and sol state (G′ = G″) represent gel‐sol transition temperature; C,D) variation of viscosity as a function of temperature; E,F) viscosity measurement of different bioinks in a shear rate sweep from 0.1 to 500 s^−1^, indicative of shear‐thinning behavior.

The gel–sol transition temperature, viscosity at the transition point and shear‐thinning behavior are important parameters of hydrogel inks.^44^ These parameters provide a reference for subsequent determination of printing schemes. The modulus‐temperature curves (**Figure** 3A,B) show that the gel–sol transition temperature of pristine GelMA7 ink (36.5 °C) is slightly lower than that of Gelatin7 ink (38.5 °C). It is possible that methacrylation modification of gelatin affects the formation of triple‐helix hydrogen bonds. The gel–sol transition temperatures of ACG35‐GelMA7 ink and ACG35‐GelMA7‐BG ink occur around at lower temperatures of 22.4 and 23 °C, indicating that the introduction of ACG further disturbs the formation of hydrogen bonds in gelatin network; while doping a small amount of BG has a negligible impact on gel–sol transition. As expected, Gelatin10 ink and GelMA10 ink respectively reveal higher gel–sol transition temperatures of 40 and 38.5 °C due to denser physical crosslinking. As described above, methacrylation leads to a slight decrease in transition temperature. After copolymerization, the gel–sol transition temperature of ACG10‐GelMA10 ink is near 29 °C; while the gel–sol transition temperature of ACG10‐GelMA10‐Mn^2+^ is identical to that of ACG10‐GelMA10 ink (data not shown), meaning that trace amount of Mn^2+^ ions has no effect on transition temperature. Compared to ACG10‐GelMA10 ink, the lower transition temperature of ACG35‐GelMA7 ink suggests that copolymerizing more of ACG leads to a disturbance to the formation of hydrogen bonds in gelatin to a greater extent.

A sufficiently large viscosity can maintain the shape fidelity of the printed filaments, and thus prevent the structures from collapsing before it is chemically crosslinked by the UV light irradiation. Figure 3C,D clearly demonstrates that the viscosities of all ACG‐GelMA inks remain stable below 22 °C, and then drop with increasing temperature. Heat‐triggered gel–sol transition is favorable for 3D printing, and a relatively high viscosity at a low temperature is advantageous for improving the fidelity of the shape of filaments and obtained scaffold. Furthermore, all the ACG‐GelMA inks exhibit similar shear thinning behaviors (Figure 3E,F), confirming that the ACG‐GelMA inks can be smoothly squeezed out of the printing nozzle.

**Figure 4:**
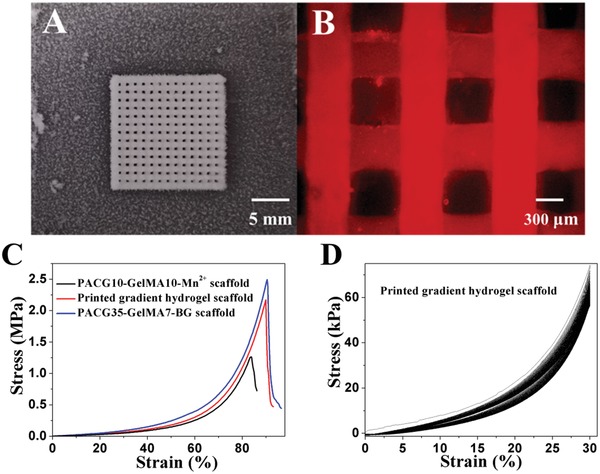
A) A photograph of ACG‐GelMA hydrogel scaffold printed by air‐extrusion method assisted with a low‐temperature receiver before UV light irradiation; B) microscope image of PACG‐GelMA hydrogel scaffold after UV light irradiation and reaching swelling equilibrium (stained with rhodamine); C) compressive stress–strain curves of the printed porous PACG10‐GelMA10‐Mn^2+^ hydrogel scaffolds, PACG35‐GelMA7‐BG hydrogel scaffolds, and gradient hydrogel scaffolds; D) cyclic compressive stress‐strain curves for the printed gradient scaffold (the top three layers were printed by PACG10‐GelMA10 hydrogel, and the bottom nine layers were printed by PACG35‐GelMA7‐BG hydrogel under a maximum strain of 30%. The cycle numbers were set as 100.

Comprehensively considering the above rheological characteristics, an air‐extrusion 3D printing method assisted with a low‐temperature receiver was adopted to precisely fabricate osteochondral regeneration scaffolds. ACG‐GelMA inks were first kept at 4 °C for 20 min to allow for full formation of triple helices in gelatin network to increase the viscosity, thus improving the printability of low concentration of GelMA bioinks.^45^ Then the printing cartridges were increased back to 20 °C to prevent the ink from excessive physical crosslinking and clogging in the printing nozzle, making sure that the stable and smooth filament could be deposited on the receiving platform controlled at −10 °C at which the shape of the fiber can be well maintained. This ensures the fidelity of the scaffold. Then the printed scaffolds were subjected to UV light irradiation in a cooling environment to initiate polymerization to eventually fix the final architecture of the scaffolds. Herein, PACG10‐GelMA10‐Mn^2+^, PACG35‐GelMA7‐BG, and gradient scaffold consisting of top cartilage layer of PACG10‐GelMA10‐Mn^2+^ and bottom bone layer of PACG35‐GelMA7‐BG were printed. **Figure** 4A,B displays the morphologies of the representative printed PACG35‐GelMA7 hydrogel scaffolds in which the interconnected macroscopic pores are orderly arranged and uniform. The scaffolds can still keep the intact geometry shape after reaching swelling equilibrium in deionized water, indicating the printed PACG‐GelMA hydrogel scaffolds can maintain a very stable swelling stability in aqueous solution, and thus structural integrity and the high resolution of the architecture. The obtained scaffolds demonstrate excellent compressive strengths (>1 MPa). The compressive strengths of PACG10‐GelMA10‐Mn^2+^ and PACG35‐GelMA7‐BG and biohybrid gradient hydrogel scaffolds are measured to be 1.24, 2.51, and 2.17 MPa, respectively. The corresponding compressive moduli are 163, 249, and 195 kPa, respectively (Figure 4C). Subjected to 100 cycles of continuous compression loading and unloading, the overlapped successive cyclic force curves can be achieved, manifesting a high elasticity of the printed scaffold (Figure 4D). Even after cyclic compression, the scaffold was able to remain its macro‐ and microstruture owing to its high strength and better elasticity.

### 2.4 Ions Release and Biofunctions of the Printed Hydrogel Scaffolds

The cumulative release behaviors of Mn^2+^ from the printed PACG10‐GelMA10‐Mn^2+^ hydrogel scaffolds and release of Sr^2+^, Si^4+^, and B^3+^ from the printed PACG35‐GelMA7‐BG hydrogel scaffolds are shown in Figure S8 in the Supporting Information. Within the initial three days, a burst release is observed owing to the rapid leakage of ions near the surface. And then these ions diffuse out of the printed porous hydrogel scaffolds gradually. Finally, ions release from the printed porous hydrogel scaffolds level off around 25 days.

Alkaline phosphatase (ALP), a well‐known marker for early osteogenic differentiation of hBMSCs, was assayed after hBMSCs cells were cultured for 7 and 14 days on PACG35‐GelMA7 and PACG35‐GelMA7‐BG hydrogel scaffolds (Figure S9, Supporting Information). The hBMSCs seeded on the PACG35‐GelMA7‐BG hydrogel scaffolds present stronger ALP activity than those on the PACG35‐GelMA7 hydrogel scaffolds and the hBMSCs on the culture plate. These results reveal that incorporation of BG can promote the osteogenic differentiation of hBMSCs.

**Figure 5:**
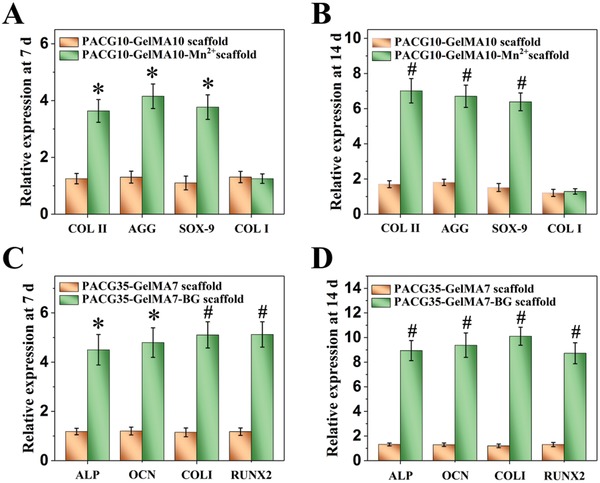
Gene analysis for chondrogenic differentiation and osteogenic differentiation from hBMSCs grown in different scaffolds. A,B) The expression of cartilage‐associated gene (COL II, aggrecan, SOX‐9, and COL I) after incubated for 7 and 14 days, respectively; C,D) the expression of osteogenesis‐associated gene (ALP, OCN, COL I, and RUNX2) after incubated for 7 and 14 days, respectively. Mn^2+^‐loaded PACG10‐GelMA10 hydrogel scaffold or BG‐loaded PACG35‐GelMA7 hydrogel scaffold significantly elevates the chondrogenic or osteogenic differentiation of hBMSCs, respectively (*p < 0.05, ^#^ *p* < 0.01, compared with control groups).

Chondrogenic and osteogenic genes expression levels at 7 and 14 days were further analyzed by real‐time quantitative polymerase chain reaction (RT‐qPCR) (**Figure** 5). For the group of PACG10‐GelMA10‐Mn^2+^ scaffolds, the expression levels of cartilage‐specific gene (COL II, AGG, and SOX‐9) are markedly upregulated compared with PACG10‐GelMA10 scaffold group at both 7 and 14 days, demonstrating that loading Mn^2+^ in the PACG10‐GelMA10 scaffold can promote the chondrogenesis. Meanwhile, there is no significant upregulation expression of fibroblastic gene (COL I) even after 14 days of culture, showing no sign of fibrocartilage formation in Mn^2+^‐doped scaffold (Figure 5A,B). Compared with PACG35‐GelMA7 scaffolds, bone‐related gene (ALP, COL I, OCN, and RUNX2) expression also displays an upregulation at both 7 and 14 days in the PACG35‐GelMA7‐BG hydrogel scaffolds, implying that continuous release of ions from the BG loaded in the PACG35‐GelMA7 hydrogel scaffolds may be conducive to enhancing the osteogenic differentiation of hBMSCs (Figure 5C,D). These results verify that combining Mn^2+^ with PACG10‐GelMA10 scaffolds and BG with PACG35‐GelMA7 scaffolds can benefit the chondrogenic and osteogenic differentiation, respectively.

### 2.5 In Vivo Osteochondral Repair Efficacy of the Gradient Hydrogel Scaffolds

**Figure 6:**
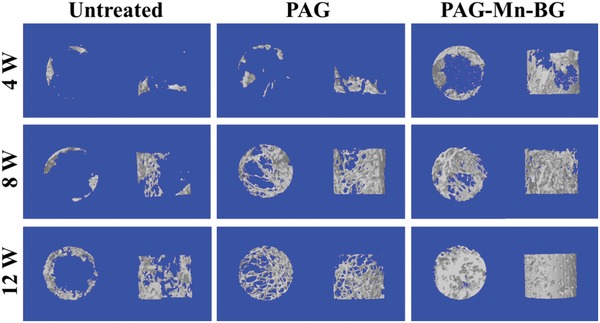
Characteristic 3D reconstruction images of micro‐CT analysis of the repaired subchondral bone at 4, 8, and 12 weeks in different groups.

To evaluate the new subchondral bone formation, micro computed tomography (micro‐CT) images were taken at 4, 8, and 12 weeks after surgery. Representative 3D reconstruction images of each group are shown in **Figure** 6. Clearly, compared with the untreated control and PAG gradient scaffold without loading Mn^2+^ or BG, the new subchondral bone formation in the PAG‐Mn‐BG (biohybrid gradient scaffold consisting of top layers of PACG10‐GelMA10‐Mn^2+^ and bottom layers of PACG35‐GelMA7‐BG) group is significantly improved at 4 weeks postimplantation. This suggests that the release of bioactive ions from the PAG‐Mn‐BG scaffold may promote the early regeneration of subchondral bone. At 8 weeks postimplantation, it is seen in the PAG scaffold group that the new tissue grows along the alignment direction of filaments of the implanted interconnected porous scaffolds. In comparison, the repair effect of subchondral bone in the PAG‐Mn‐BG scaffold group is further improved. Interestingly, the newly formed subchondral bone is especially pronounced in the PAG‐Mn‐BG scaffold group at 12 weeks postimplantation, where the entire defect area is filled with new subchondral bone. The repair effect of subchondral bone is also improved in the PAG scaffold group, but to a lesser extent than in the PAG‐Mn‐BG scaffold group, since there is still a large cavity in the defect region. In contrast, the defects in the untreated group show only very little new subchondral bone formation along the edge of the implantation. All these results prove that the 3D printed stiff gradient hydrogel scaffold can play an important role in template‐guide and mechanical support, and can further speed up the regeneration of subchondral bone after the introduction of ions. Quantitative analysis data of the newly formed subchondral bone within the defect further confirm the micro‐CT findings (Figure S10, Supporting Information). For all the three groups, the values of the ratio of bone volume to tissue volume (BV/TV) and bone mineral density (BMD) in trabecular volume of interest (VOI) keep increasing with the extension of implantation time, but the trend of increase varies considerably. Both values are significantly higher in the PAG‐Mn‐BG scaffold group than in the untreated and PAG scaffold groups at the same postimplantation time, indicating that the PAG‐Mn‐BG scaffold containing various bioactive ions can boost osteochondral tissue repair in the defects.

**Figure 7:**
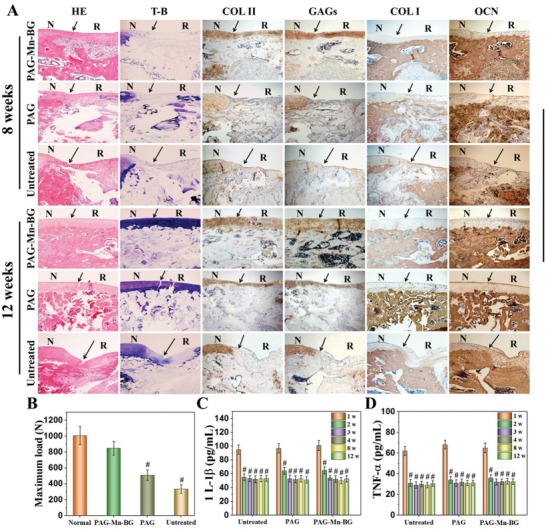
Histological assessment of repaired cartilage subchondral bone at 8 and 12 weeks postsurgery in different groups. A) Hematoxylin and eosin (H&E), toluidine blue (T‐B) staining, and immunohistological staining for Coll II, GAGs, COL I, and OCN, show simultaneously enhanced cartilage and subchondral bone repair in the PAG‐Mn‐BG group, compared to the PAG group and the untreated control (blank) (N: normal cartilage; R: repair cartilage; the arrows indicate the margins of the normal cartilage and repaired cartilage; Scale bar = 200 µm); B) compression destruction tests of the repaired knees at 12 weeks postsurgery in different groups (^#^ *p* < 0.01, compared with the normal knees); C,D) quantification analysis of interleukin‐1 (1L‐1β) and tumor necrosis factor‐α (TNF‐α) in the serum of experimental rats at 1, 2, 3, 4, 8, and 12 weeks after surgery (^#^ *p* < 0.01, compared with 1 week at the same group).

Histological staining analysis further confirms that the PAG‐Mn‐BG scaffold is able to simultaneously enhance the repair of articular cartilage and subchondral bone, relative to the PAG scaffold and the untreated control (**Figure** 7A). As shown in hematoxylin and eosin (H&E) and toluidine blue (T‐B) staining, new cartilage and bone in the PAG‐Mn‐BG scaffold group are observed at the edges of the defects at 8 weeks postimplantation. At 12 weeks postimplantation, a layer of new cartilage is observed in the chondral region treated with PAG‐Mn‐BG group, with a thickness similar to that of the adjacent cartilage. Similarly, the subchondral bone area is also filled with new bone. Both regenerated cartilage and subchondral bone are well integrated with the host tissues. In comparison, the defects treated with the PAG scaffold show less effective bone and cartilage repair with poor quality even at 12 weeks; while empty cavities and scarce regenerated tissue are present in the untreated defects, with the collapse of adjacent cartilage. Immunohistochemical (IHC) staining for both cartilage and bone‐specific proteins were also carried out to further evaluate the osteochondral repair. COL II and GAGs are positively and uniformly stained in PAG‐Mn‐BG scaffold group, and the protein expressions are increased with time. It is worth noting that the staining for these two proteins are much stronger in the PAG‐Mn‐BG scaffold group than in the PAG and the untreated control groups especially after 12 weeks postimplantation. Both proteins of COL I and OCN are positively stained at the subchondral bone region in the group treated with PAG‐Mn‐BG scaffold, with a time‐dependent increase in staining intensity, and remarkably stronger than those in the PAG and the untreated control groups. Furthermore, there is negligible COL I or OCN distributed in neo‐cartilage layer. These above results indicate that the 3D printed PAG‐Mn‐BG scaffold can simultaneously enhance cartilage and subchondral bone repair, benefiting from the force support at early stage and the function of various ions released continuously from the 3D printed PAG‐Mn‐BG scaffold and the degradation characteristics of the 3D printed PAG‐Mn‐BG scaffold.

High‐resolution scanning electron microscope (SEM) is further used to assess microstructure of the repaired cartilage (Figure S11, Supporting Information). Interestingly, at 12 weeks postsurgery, the microtopography of the repaired cartilage treated with PAG‐Mn‐BG scaffold is smooth and uniform, and has no noticeable difference with the normal cartilage. On the contrary, the surface is rough and even some cracks appear on the repaired tissues in the PAG scaffold group and untreated defect group.

Figure 7B shows the compression destruction test of the repaired knees at 12 weeks postsurgery. For the knees treated with PAG‐BG‐Mn scaffold, the value of maximum failure load is close to that of the normal knees, and significantly higher than those of the knees treated with PAG scaffold and untreated control groups. These results are in accordance with micro‐CT and histological staining analysis results.

Inflammatory cytokines in the serum of experimental rats were quantitatively analyzed (Figure 7C,D). All three groups present an initial increase in cytokines during the acute phase at 1 week after surgery. This is normal acute inflammatory reaction;^3^ however, the levels of both inflammatory factors maintained a relatively low level during the repair process from 2 to 12 weeks. Although we did not make a comparison with the healthy normal group, the decrease in the levels of main inflammatory factors indirectly reflected that the inflammatory response was decreased with time. Collectively, the PAG‐Mn‐BG scaffold recapitulating osteochondral architecture and niche can efficiently accelerate the concurrent regeneration of cartilage and subchondral bone.

## 3 Conclusions

Conventional gelatin hydrogels are poor in mechanical properties, precluding their applications as load‐bearing scaffolds. In this study, a novel biodegradable and supramolecular hydrogen bonding strengthened chemical crosslinked gelatin hydrogels were successfully prepared by copolymerization of ACG and GelMA. The cleavable dynamic hydrogen bonds of PACG could considerably strengthen and stiffen the inherently weak GelMA hydrogel to possess a high compressive strength (up to 12.4 MPa),and compressive modulus (up to 837 kPa), much superior to those of reported GelMA hydrogels (≈200 kPa compressive strength and ≈100 kPa compressive modulus) without undermining its thermosensitive printability, and in turn, the chemical crosslinking of GelMA served to stabilize the intrinsically transient PACG network. The first reported high strength gelatin hydogel can provide a mechanical support in the early stage of osteochondral repair. In mimicking articular cartilage‐subchondral bone architecture, a bilayer biohybrid gradient hydrogel scaffold consisting of top cartilage layer of PACG‐GelMA‐Mn^2+^ and bottom bone layer of PACG‐GelMA loaded with bioactive glass was precisely tailored by one‐step thermal‐assisted extrusion printing technique, followed by UV light irradiation to initiate polymerization at a cooled temperature to fix the formed high strength construct. Incorporating BG could improve the proliferation, ALP activities and differentiation of hBMSCs, and loading Mn^2+^ facilitated chondrogenic differentiation of the hBMSCs. The resultant biohybrid gradient hydrogel scaffold showed superior performance for accelerating cartilage and subchondral bone repair simultaneously in rat knee osteochondral defect. It is our belief that this 3D printed high‐strength and biodegradable biohybrid gradient hydrogel scaffold can be extended to treatment of other load‐bearing tissue defects. Of course, the present system cannot be directly printed with cells since small molecular ACG monomer was mixed with GelMA to produce a ink. We believe this strategy can be extended to enhance the mechanical strength of other naturally occurring biomacromolecule hydrogels.

## Conflict of Interest

The authors declare no conflict of interest.

## Supporting information

Supplementary
