# Supplementary material for: Osteochondral Regeneration with 3D‐Printed Biodegradable High‐Strength Supramolecular Polymer Reinforced‐Gelatin Hydrogel Scaffolds
Source: Adv Sci (Weinh). 2019 Jun 11;6(15):1900867. doi: 10.1002/advs.201900867 (PMC6685475; doi:10.1002/advs.201900867)
Supplement: Supplementary file 1 — Supplementary [file ADVS-6-1900867-s001.pdf]

Copyright WILEY-VCH Verlag GmbH & Co. KGaA, 69469 Weinheim, Germany, 2019.

Supporting Information

for *Adv. Sci.*, DOI: 10.1002/advs.201900867

Osteochondral Regeneration with 3D-Printed Biodegradable High-Strength Supramolecular Polymer Reinforced-Gelatin Hydrogel Scaffolds

Fei Gao, Ziyang Xu, Qingfei Liang, Haofei Li, Liuqi Peng, Mingming Wu, Xiaoli Zhao, Xu Cui, Changshun Ruan, and Wenguang Liu**

Osteochondral Regeneration with 3D-Printed Biodegradable High-Strength Supramolecular Polymer Reinforced-Gelatin Hydrogel Scaffolds

Fei Gao[1], Ziyang Xu[1], Qingfei Liang[2], Haofei Li[1], Liuqi Peng[2], Mingming Wu[2], Xiaoli Zhao[2], Xu Cui[2], Changshun Ruan*[2], Wenguang Liu*[1]

[1]School of Materials Science and Engineering, Tianjin Key Laboratory of Composite and Functional Materials, Tianjin University, Tianjin 300350, China

[2]Research Center for Human Tissue and Organs Degeneration, Institute Biomedical and Biotechnology, Shenzhen Institutes of Advanced Technology, Chinese Academy of Sciences, Shenzhen 518055, China

*Corresponding author (E-mail: wgliu@tju.edu.cn, cs.ruan@siat.ac.cn)

Experimental Section

Materials.

Glycine (98%, TCI, Shanghai, China), acryloyl chloride (98%, TCI, Shanghai, China), gelatin from porcine skin (type A, reagent grade, Sigma-Aldrich), methacrylic anhydride (94%, Heowns Biochemical Technology Company, China), collagenase (Solarbio Science & Technology Co., Ltd., China), 2-hydroxy-2-methyl-1-phenyl-1-propanone (IRGACURE 1173, 98%, Sigma-Aldrich, St. Louis, USA), and $MnCl_2$ (98%, TCI, Shanghai, China) were used as received. Bioglass ($6Na_2O \cdot 8K_2O \cdot 8MgO \cdot 16CaO \cdot 6SrO \cdot 27B_2O_3 \cdot 27SiO_2 \cdot 2P_2O_5$) was prepared as previously reported protocol.[1] N-acryloyl 2-glycine (ACG) was synthesized according to our previous study.[2] All other chemical reagents (sodium hydroxide, hydrochloric acid, N, N-dimethylformamide, ethyl acetate and dichloromethane, Tianjin Jindongtianzheng Precision Chemical Reagent Factory, China) were of analytical grade.

Synthesis of Methacrylated Gelatin (GelMA).

GelMA was synthesized according to a previously described method. [3] Briefly, 4 g type A porcine skin gelatin was dissolved in 200 mL deionized water at 40 °C with stirring. The solution pH was adjusted by adding sodium hydroxide dropwise to 7.4 and stirred until it became homogeneous and clear. Then 80 mL dimethyl formamide was added. Subsequently 300 μL methacrylic anhydride was slowly added at a speed of 0.5 mL min^{-1} and the reaction proceeded at 40 °C for 2 h. The mixture was precipitated with ethanol, and the collected precipitate was then dissolved in deionized water to dialysis. The molecular weight cut-off (MWCO) of the dialysis was 3500 and the time of the dialysis lasted three days. Finally, the purified solution was lyophilized to obtain the resultant GelMA.

Determination of the Degree of Methacrylation (DM).

The DM of GelMA was determined by integration of ^1H NMR spectra. The gelatin

and GelMA were dissolved in D_2O at a concentration of 10 mg/mL. Three spectra were recorded for each sample. The spectra were normalized to the phenylalanine signal (6.9-7.5 ppm), which represented the concentration of gelatin. The DM of GelMA was calculated as equation (1)：[4]

$$DM(\%) = (1 - \frac{A(\text{lysine methylene of GelMA})}{A(\text{lysine methylene of gelatin})}) \times 100\% \qquad (1)$$

Where, A(lysine methylene of gelatin) and A(lysine methylene of GelMA) are the integrated intensities of lysine methylene at 2.8-2.95 ppm in the spectra of unmodified gelatin and GelMA, respectively.

Preparation of PACG-GelMA, PACG-GelMA-Mn^{2+} and PACG-GelMA-BG Hydrogels.

PACG-GelMA copolymer hydrogels were prepared by photo-initiated radical copolymerization. Briefly, an appropriate mass of ACG and GelMA was first dissolved in deionized water according to the designed formulations. Then 1 wt% photoinitiator IRGACURE 1173 (relative to the total weight of monomers) was added into the solution. Subsequently, the mixture was cast into plastic rectangle molds (length 100 mm, width 50 mm, thickness 0.5 mm) or plastic tubular molds (inner diameter 10 mm, length 10 mm), and the polymerization was carried out for 40 min in a crosslink oven (XL-1000 UV Crosslinker, Spectronics Corporation, NY, USA). A series of hydrogels were prepared by varying initial ACG or GelMA monomer concentrations in this study. To simplify the discussion, the obtained hydrogels were coded as PACGX-GelMAY, X and Y represented the initial mass percentage concentration of ACG and GelMA, respectively.

For PACG-GelMA-Mn^{2+} hydrogels, we first prepared a series of $MnCl_2$ aqueous solutions with different concentrations (0.125, 0.25, 0.5, 1, 2, 4 ppm) and added a certain amount of ACG and GelMA to $MnCl_2$ aqueous solutions. After uniform dissolution, the PACG-GelMA hydrogel doped with Mn^{2+} was formed by UV light irradiated polymerization. Similarly, for PACG-GelMA-BG hydrogels, a certain amount of BG powder was added into the solution of ACG and GelMA. After uniform

dispersion, the PACG-GelMA hydrogel loading BG was obtained by UV light irradiated polymerization. Herein, the contents of BG accounting for 1%, 3% and 5% of the total mass of ACG and GelMA were selected to make hybrid hydrogels.

Characterizations of Hydrogels.

FTIR Spectroscopy: Fourier transform infrared (FTIR) spectrometry (PerkinElmer spectrum 100, USA) was used to characterize the chemical structure of the GelMA, the formation of PACG-GelMA hydrogel, and the successful doping of BG into the PACG-GelMA Hydrogels.

Dynamic Swelling Behaviors and Equilibrium Water Contents (EWCs) of PACG-GelMA Hydrogels: PACG-GelMA hydrogels with varied initial monomer concentrations of ACG and GelMA were prepared in form of cylinder with a diameter of 4.4 mm and a height of 5 mm. To detect the dynamic swelling behaviors, the freshly prepared gels were weighed on a microbalance to record their initial wet weights (W_i), and then separately immersed in deionized water at 37 ºC. At specified time intervals, the gels were removed from the medium, and the weight of the hydrogel in swollen state (W_t) was recorded quickly after the excess water on the surface was gently blotted. The swelling ratio of the hydrogels was calculated as equation (2) :

$$SR = \frac{W_t}{W_i} \qquad (2)$$

Where W_t is the wet weight of the swollen hydrogels at time t (t is the time the hydrogel was immersed in deionized water), and W_i is the initial wet weight of the hydrogels. All experiments were performed in triplicate. The equilibrium water contents (EWCs) of the hydrogels were determined as the reported method.[2]

***In Vitro* Degradation of PACG-GelMA Hydrogels:** The degradation behaviors of the PACG-GelMA hydrogels with varying initial monomer concentrations of ACG and GelMA were determined by an enzymatic degradation process. Circular samples with 8 mm diameter and 1 mm thickness were incubated in a freshly prepared collagenase II solution (2 μg/mL) in PBS at 37 ºC in a reciprocal shaking incubator at

60 rpm. The media was added with 50 μg/mL gentamycin to prevent the growth of microorganisms. The solution was renewed every three days. At predetermined time intervals, the hydrogels were removed from the incubation medium, exhaustively washed with deionized water, then freeze-dried and weighed. The percent remaining mass was calculated as equation (3) : [5]

$$D\%=\frac{W_{o,\,dry}-W_{t,\,dry}}{W_{o,\,dry}}\times 100\% \qquad (3)$$

where $W_{o,\,dry}$ is the initial dry weight of the sample, and $W_{t,\,dry}$ is the dry weight at each incubated time point.

Mechanical Properties of PACG-GelMA, PACG-GelMA-Mn^{2+} and PACG-GelMA-BG Hydrogels: Mechanical tests were performed at room temperature after an equilibrium swelling state was achieved in deionized water using a WDW-05 electromechanical tester (Time Group Inc., China). Dumbbell shape samples with 2 mm in width, 10 mm in gauge length were used for tensile tests. The rate of extension was fixed at 50 mm min^{-1}. Cylindrical samples (diameter 4 mm, height 5 mm) were used for compression tests. The crosshead speed was set at 10 mm min^{-1}. At least eight samples were used in each mechanical test.

Rheological Test of Hydrogels: Different rheological characteristics of PACG10-GelMA10-Mn^{2+} and PACG35-GelMA7-BG hydrogels were carried out on a rheometer (HAKKE MASE Ш, Germany). For frequency sweep tests, with σ = 20 Pa, T = 30 ºC, the frequency range was set from 0.1 to 20 Hz (equivalent to 0.63-126 rad/s). And for cyclic alternating shear strain sweep tests**,** with T = 30 ºC, f = 1Hz, the shear strain was changed successively from strain= 10% (50 s) to strain= 100%/300% (50 s), and the cycle was repeated for three times, and then 50 s was tested under the action of 10% strain. After each phase of the test, the next phase was immediately switched without retention. At least three effective samples were tested for each hydrogel sample.

Rheological Test of Inks: Rheological characteristics of Gelatin7, GelMA7, GelMA10, ACG10-GelMA10, ACG35-GelMA7, ACG10-GelMA10-Mn^{2+} and ACG35-GelMA7-BG inks prior to UV light irradiation were also tested. For

temperature sweep tests, a suitable amount of ink was put on the rheometer and cooled to 4 ºC to form gel state, and then the storage modulus G', loss modulus G' and the viscosity over a temperature range of 10 - 40 ºC were recorded with a heating rate of 2 ºC/min (σ = 20 Pa, f = 1 Hz). For shear rate sweep tests, shear viscosity was recorded over a shear rate range of 0.1 to 500 s^{-1}.

Cell Proliferation Assay: A cell counting kit-8 (CCK-8, Beyotime, China) was used to evaluate the effect of Mn^{2+} or BG doping on cell proliferation at 1, 4 and 7 days, respectively. Considering that the ions could be lost during the process of soaking deionized water to reach equilibrium swelling. Therefore, we directly cultured cells with a-MEM containing different Mn^{2+} concentrations (0.125, 0.25, 0.5, 1, 2, 4 ppm) or an extract of PACG35-GelMA7-BG hydrogel (40 mg in 1 mLα-MEM). The viability of the human bone marrow mesenchymal stem cells (hBMSCs, HUXMA-01001, Cyagen, China) seeded on PACG10-GelMA10 hydrogel cultured for 24 h with or without 0.5 ppm Mn^{2+} and on PACG35-GelMA7 hydrogel cultured for 24 h with or without the extract of PACG35-GelMA7-BG was assessed by a Live-Dead staining kit (CFSE, Dojindo Laboratorise, Japan) according to manufacturer’s guidelines. Live cells were stained green by intracellular esterase-catalyzed hydrolysis of Calcein AM and dead cells were stained red by ethidium homodimer-1 by penetrating through the damaged membranes and binding to nucleic acids. The cellular morphology was observed qualitatively on an OLYMPUS CKX41 fluorescence microscope (OLYMPUS, Japan).

3D Printing of Biohybrid Gradient PACG-GelMA Hydrogel Scaffolds.

Hydrogel scaffolds were produced by a bioplotter pneumatic dispensing system (BioScaffolder 2.1, GeSiM, Grosserkmannsdorf, Germany), which pneumatically deposited hydrogel strands onto a stationary platform that was set at -10 ºC. Briefly, the hydrogel ink was filtered by 0.22 mm filter and loaded into a sterile cartridge, incubated for 20 min at 4 ºC to increase the ink viscosity. Then, the pre-hydrogel strands were plotted into a glass slides at a plotting speed of 15 mm/s, under a

pressure of about 55 kPa for ACG10-GelMA10 and ACG10-GelMA10-Mn^{2+}, 45 kPa for ACG35-GelMA7 and ACG10-GelMA10-BG through a conical needle with inside diameter of 350 μm at 20 ºC, respectively. Each layer adhered to the underlying layer perpendicularly to form a 0°/90° structure, and the spacing between each strand was set at 1.1 mm. The PACG10-GelMA10, PACG10-GelMA10-Mn^{2+}, PACG35-GelMA7 and PACG35-GelMA7-BG hydrogel scaffolds were obtained after UV light-irradiated crosslinking for 10 min in a cooled crosslink oven.

Two-channel alternate printing technique was adopted to fabricate the biohybrid gradient hydrogel scaffolds with Mn^{2+} being loaded on the top three layers and BG being uniformly incorporated on the bottom nine layers. The sterile ACG10-GelMA10-Mn^{2+}, ACG35-GelMA7-BG inks were filled into two cartridges, respectively, and incubated for 20 min at 4 ºC. Nine layers of ACG35-GelMA7-BG pre-hydrogel were printed firstly, then three ACG10-GelMA10-Mn^{2+} pre-hydrogel layers were printed continually. A 12-layers of gradient hydrogel scaffold was fabricated after post-crosslinking with UV light irradiation.

Characterization of Biohybrid Gradient PACG-GelMA Hydrogel Scaffolds.

Mechanical Properties of PACG-GelMA Hydrogel Scaffolds: The mechanical properties of the 3D-printed porous PACG10-GelMA10-Mn^{2+}, PACG35/GelMA7-BG and the gradient PACG-GelMA-Mn^{2+}-BG hydrogel scaffolds were evaluated through a uniaxial compression test. After reaching equilibrium swelling in deionized water, the scaffolds were cut into cylinders (5 mm in diameter and 3.3 mm in height) and measured as the aforementioned method. The cyclic compression experiment of the printed gradient hydrogel scaffolds (5 mm in diameter and 3.3 mm in height) was also performed. The compressive loading and unloading were repeated 100 cycles with a 30% displacement of gel height at room temperature. At least eight samples were used for each test.

Ion Leaching Analysis: The Mn^{2+} release from PACG10-GelMA10-Mn^{2+} hydrogel scaffolds and release of Sr^{2+}, Si^{4+}, and B^{3+} release from PACG35-GelMA7-BG

hydrogel scaffolds were characterized as follows. The printed scaffolds (10×10 mm, 6 layer) were immersed into 3 mL deionized water and shaken at 60 rpm at 37 °C. At different immersion time points (0.5, 1, 2, 3, 5, 7, 10, 14, 19, and 25 days), 500 μL solution was taken out and corresponding 500 μL fresh deionized water was supplemented. The collected solution was appropriately diluted, and analyzed by inductively-coupled plasma optical emission spectrometry (Perkin Elmer, Optima 7000DV, Massachusetts, USA).

Alkaline Phosphatase (ALP) Activity Test: In order to investigate the effect of incorporated BG on the differentiation of hBMSCs, the expression of ALP in the hBMSC cultured with or without the extract (40 mg in 1 mL α-MEM) of PACG35-GelMA7-BG hydrogel scaffolds for 7 and 14 d were quantified by using Amplite Colorimetric Alkaline Phosphatase Assay Kit (AAT Bioquest) according to the manufecturer’s protocol. hBMSCs were seeded with an initial density of 2×10^5 on each scaffold in 24-well cell culture plates containing 750 μL of α-MEM (Gibco, USA) supplemented with 10% fetal bovine serum (FBS), 100 U/mL penicillin, and 100 μg/mL streptomycin. The cell-seeded scaffolds were cultivated in an incubator at 37 °C and 5% CO_2, and the cell culture medium was replaced every two days. After 7 and 14 days of culture, the scaffolds seeded with hBMSCs cells were rinsed thrice with PBS and then lysed in 500 μL lysis buffer (RIPA buffer, Beyotime Biotechnology, Shanghai, China) at 4 °C for 30 min. After centrifugation for 4 min at 2000 rpm and 4 °C to remove cell debris, 50 μL of the supernatant was added to 50 μL chromogenic substrate and incubated at 37 °C for 30 min. The reaction was stopped by adding 100 μL stop buffer. Absorbance was measured at 405 nm using a microplate reader. ALP levels were normalized to the total protein content, which was determined by a commercially available protein assay kit (Pierce™ BCA Protein Assay Kit, Thermo Fisher Scientific, Massachusetts, USA), read at 595 nm and calculated according to a series of albumin (BSA) standards.

Quantitative Real-Time Polymerase Chain Reaction (qRT-PCR) Analysis: After respectively culturing with the extracts of PACG35-GelMA7-BG hydrogel scaffolds (40 mg in 1 mL α-MEM, the hBMSCs seeded on PACG35-GelMA7 hydrogel

scaffolds) and 0.5 ppm Mn^{2+} (the hBMSCs seeded on PACG10-GelMA10 hydrogel scaffolds) for 7 and 14 days, the expression of osteogenic specific genes (ALP, COL I, OCN and RUNX2) and chondrogenic marker genes (COL II, AGG, SOX-9 and COL I) were analyzed by qRT-PCR using an ABI 7500 RT-PCR system (Applied Biosystems, Massachusetts, USA) with SYBR Green PCR Master Mix (Toyobo Life Science, Osaka, Japan) as previously described. The specimens were washed twice with PBS buffer and lysed in TRIZOL (Invitrogen, California, USA). Total RNA was isolated and reverse transcribed (2 μg) to cDNA using High-capacity cDNA Reverse Transcription kit (Applied Biosystem). The conditions of RT-PCR were as follows: 95 °C for 2 min, followed by 40 cycles of 95 °C for 15 s, 60 °C for 15 s and 72 °C for 30 s. The house-keeping gene β-actin was used to normalize the relative mRNA expression level of each gene using the $2^{-\Delta\Delta Ct}$ method. The relative gene amount of CTR group at day 7 was set as 1. The real-time PCR primers were synthesized as shown in **Table S1**.

***In Vivo* Osteochondral Repair and Evaluation.**

Surgical Procedure and Scaffold Implantation: A rat osteochondral defect model was created to evaluate the repair effect of the printed PACG-GelMA hydrogel scaffolds and biohybrid gradient PACG-GelMA hydrogel scaffolds *in vivo*. A total of 45 male Sprague Dawley rats (12-weeks-old) were used in this study. Briefly, the animals were anesthetized by isoflurane, and randomly divided into three groups, namely biohybrid gradient PACG-GelMA hydrogel scaffold (named as PAG-Mn-BG), pristine PACG-GelMA hydrogel scaffold (named as PAG) and untreated control group. An osteochondral defect (2.5 mm in diameter and 3.3 mm in depth) was created using a sterile drill in the center of the trochlear groove on each knee of the rats. Subsequently the as-printed porous scaffolds were implanted into the defects, while the defects in control group were left untreated. Then the incisions were closed with a degradable suture, and the rats were housed in ventilated rooms and given access to water and food. All procedures for animal experiments were approved by the Animal Care Committee of Shenzhen Institute of Advanced Technology, Chinese

Academy of Science. All experiments were conducted in accordance with the guidelines of the local Animal Welfare Committee.

Micro Computed Tomography (micro-CT) Analysis: At 4, 8, 12 weeks of post-implantation, the rats (n = 5 in each group) were euthanized, and the femoral was explanted and fixed in 4% (w/v) buffered paraformaldehyde. The new subchondral bone formed in the osteochondral defect was assessed using a micro-CT system (SkyScan 1172, Bruker, Belgium). Scanning parameters used were as follows: 18 μm resolution, 0.2 mm aluminum filter, 80 kV voltage, and 112 μA current. 3D reconstructions were made using mimics software provided by the company (CTVol). The lower/upper grey threshold was set at 85/255, respectively. the ratio of bone volume to tissue volume (BV/TV) and bone mineral density (BMD) were quantitatively determined.

Histological Evaluation: After the micro-CT analysis, the samples were decalcified in 15% (w/v) ethylenediamine-tetra acetic acid (EDTA), dehydrated through series of ethanol, embedded in paraffin, and longitudinally sectioned into slices with an approximate thickness of 5 μm using a paraffin microtome (Leica EG 1160). The sections were subsequently stained with hematoxylin and eosin (H&E), toluidine blue (T-B) and immunohistochemical (IHC) staining (including collagen type II (COL II), glycosaminoglycans (GAGs), collagen type I (COL I) and osteocalcin (OCN)) to observe new tissues. Specific, the sections were immersed in PBS containing 3% (w/v) H_2O_2 at 25 ºC for 25 min to block nonspecific reactions, and then blocked in 3% (v/v) BSA solution. Subsequently, the sections were incubated with primary antibodies against COL II (NBP2-33343, Novus), GAGs (GB11373, Servicebio), COL I (NB600-450, Novus), and OCN (ab13418, Abcam). After washing with PBS three times, the samples were incubated in 3, 3-diaminobenzidiine tetrahydrochloride (DAB) for visualization, and nuclei were counterstained with hematoxylin. Images were taken using an Olympus IX71 microscope (IX71, Japan).

Morphological Examination of Repaired Cartilage: The morphologies of the repaired cartilage at 12 weeks in untreated defect group, PAG group and PAG-Mn-BG group were examined using a scanning electron microscope (SEM, s4800, Hitachi Ltd., Japan). Briefly, the area of repaired cartilage was carefully harvested and fixed

in 4% (w/v) buffered paraformaldehyde for 2 days, dehydrated with a graded series of 70%, 80%, 90%, 95% and 100% ethanol, dried in a vacuum drying oven, and then coated with gold using a high-vacuum gold spatter coater before examination.

Compression Destruction Test: Compression destruction tests of the repaired knees were performed using a WDW-05 electromechanical tester (Time Group Inc., China). After 12 weeks post-surgery, the knees of each animal were harvested, the soft tissue on the surface of the knee joint was carefully dissected, and the posterior cortex and sparse cancellous bone tissue of the distal femur were also resected with a chainsaw. This compression destruction tests were carried out within 5 h after sacrificing rats.

Inflammatory Cytokines Analysis: Blood was extracted from the external jugular vein at various predetermined time points (1, 2, 3, 4, 8, 12 weeks) and serum was obtained after blood samples clotted for 2 hours at room temperature. The concentrations of Interleukin-1 (IL-1) and tumor necrosis factor-a (TNF-a) were quantitatively detected using standard ELISAs (Rat IL-1 beta ELISA Kit, RL800; Rat TNF-a ELISA Kit, RTA00; R&D systems) according to the manufacturer’s protocol.

Statistical Analysis

Data are expressed as mean ± standard deviation (SD). All the experiments were analyzed by one-way analysis of variance (ANOVA) with Tukey’ post hoc test. For all testing, the level of significance was set at $^{*}p < 0.05$ and $^{\#}p < 0.01$.

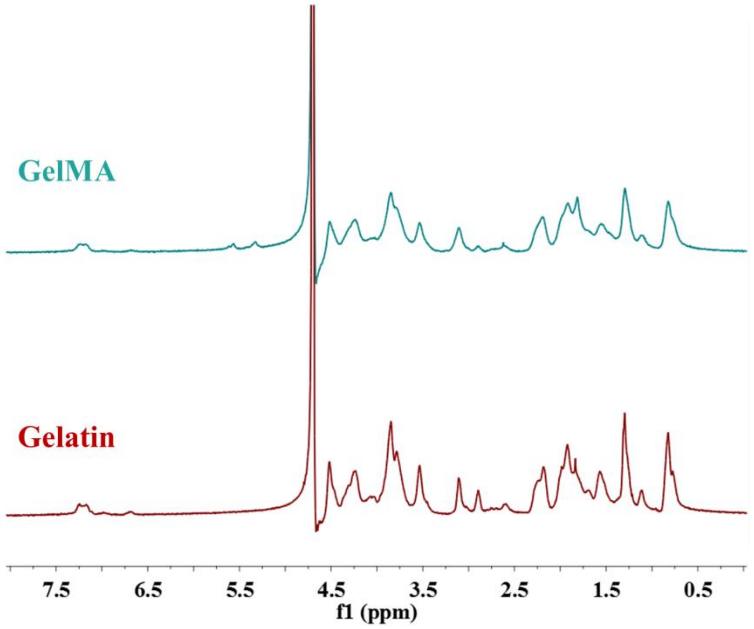

Figure S1. ^{1}H NMR spectra of gelatin and GelMA in D_2O at room temperature.

Figure S1 shows ^{1}H NMR spectra of unmodified gelatin and GelMA samples. Comparing the spectra of gelatin and GelMA, new signals that appear at 5.3 ppm and 5.5 ppm are assigned to the acrylic protons of methacrylic functions, and a new signal at 1.8 ppm is ascribed to the methyl of the introduced methacrylate. While the signal from lysine methylene at 2.9 ppm in the spectra of GelMA is decreased compared to that of gelatin spectrum.[4] These results indicate that gelatin has been successfully modified by grafting the MA. The quantitative analysis of the ^{1}H NMR spectra shows that the degree of methacrylation of gelatin was 29%.

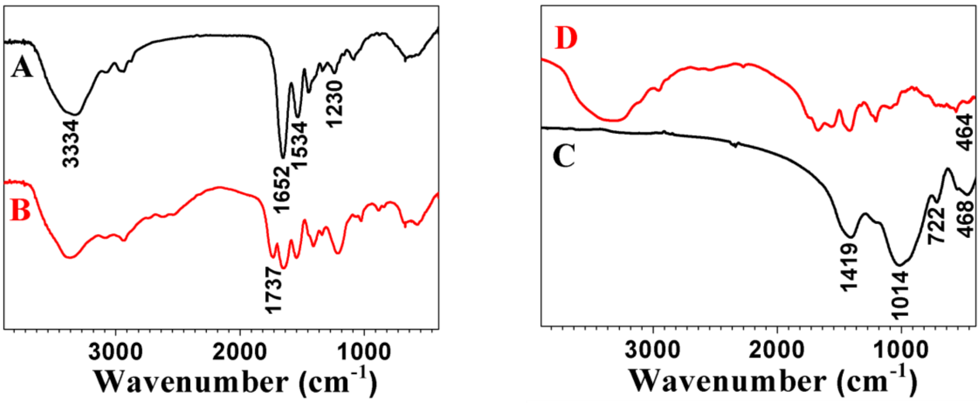

Figure S2. FTIR spectra of GelMA (A) and PACG-GelMA hydrogel (B), BG (C) and PACG-GelMA-BG hydrogel (D).

Figure S2 shows FTIR spectra of the hydrogels and BG. The feature peaks from GelMA (A) display those of gelatin backbone. The strong absorption peak at 3334 cm^{-1} is ascribed to the stretching vibration of N-H bands (amide A), the characteristic peaks at 1652 cm^{-1} is assigned to the stretching vibration of C=O (amide I), the characteristic bands at 1534 cm^{-1} is attributed to the stretching vibration of N-H bands (amide II), and the characteristic peaks at 1230 cm^{-1} is ascribed to C-N stretching vibrations of the amino acid side-chains (amide III).[6] In the spectrum of PACG-GelMA hydrogel (B), the characteristic peak at 1737 cm^{-1} is assigned to C=O stretching of carboxyl groups in the side of the PACG chain.[2] While the peaks of N-H bands, C-O stretching bands, and C-N stretching vibrations ascribed to ACG are overlapped with those belonging to the GelMA. The spectrum of BG (C) shows peaks at 468, 722, 1014 and 1419 cm^{-1} that correspond to Si-O-Si and P-O bonds, respectively. [7] The FTIR spectrum of PACG-GelMA-BG hydrogel (D) was similar to that of PACG-GelMA hydrogel, and only a new peak at 464 cm^{-1} corresponding to Si-O-Si bond can prove the successful incorporation of BG particles into the copolymer hydrogel.

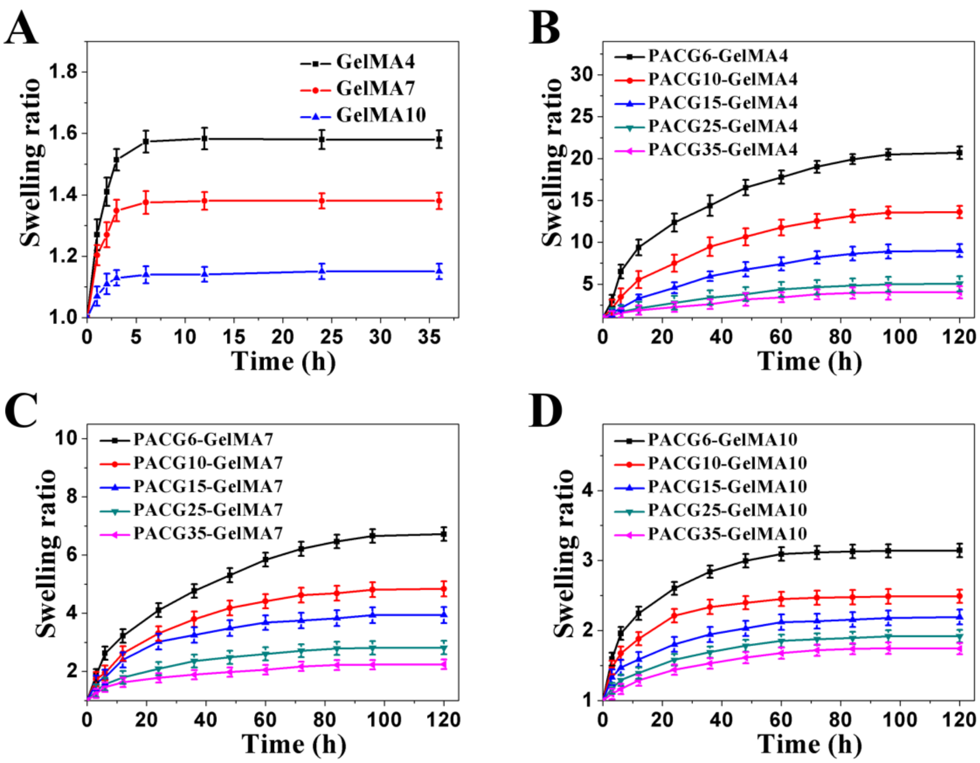

Figure S3. Dynamic swelling behaviors of PACGX-GelMAY hydrogels with varied initial concentrations of ACG and GelMA in deionized water.

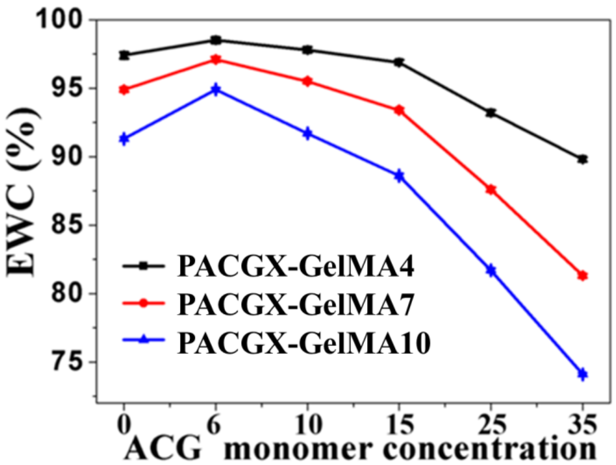

Figure S4. EWCs of PACGX-GelMAY hydrogels with varied initial concentrations of ACG and GelMA.

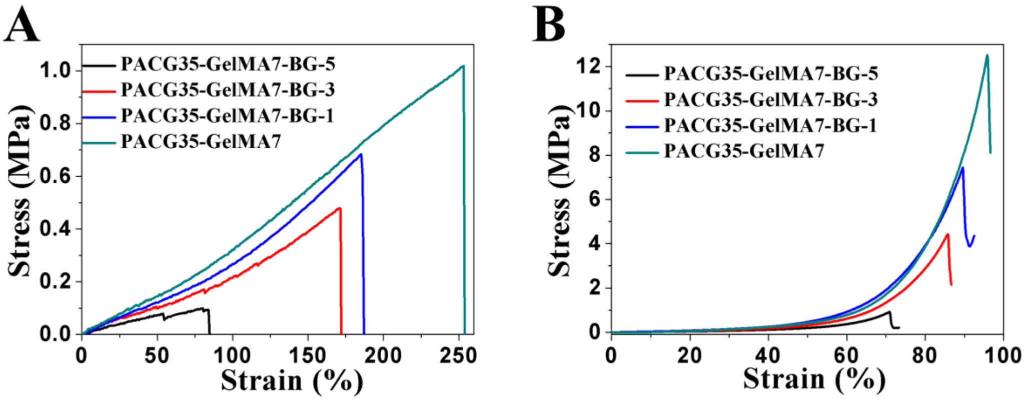

Figure S5. Tensile stress-strain curves (A) and compressive stress-strain curves (B) of the PACG35-GelMA7 hydrogels with varied BG contents.

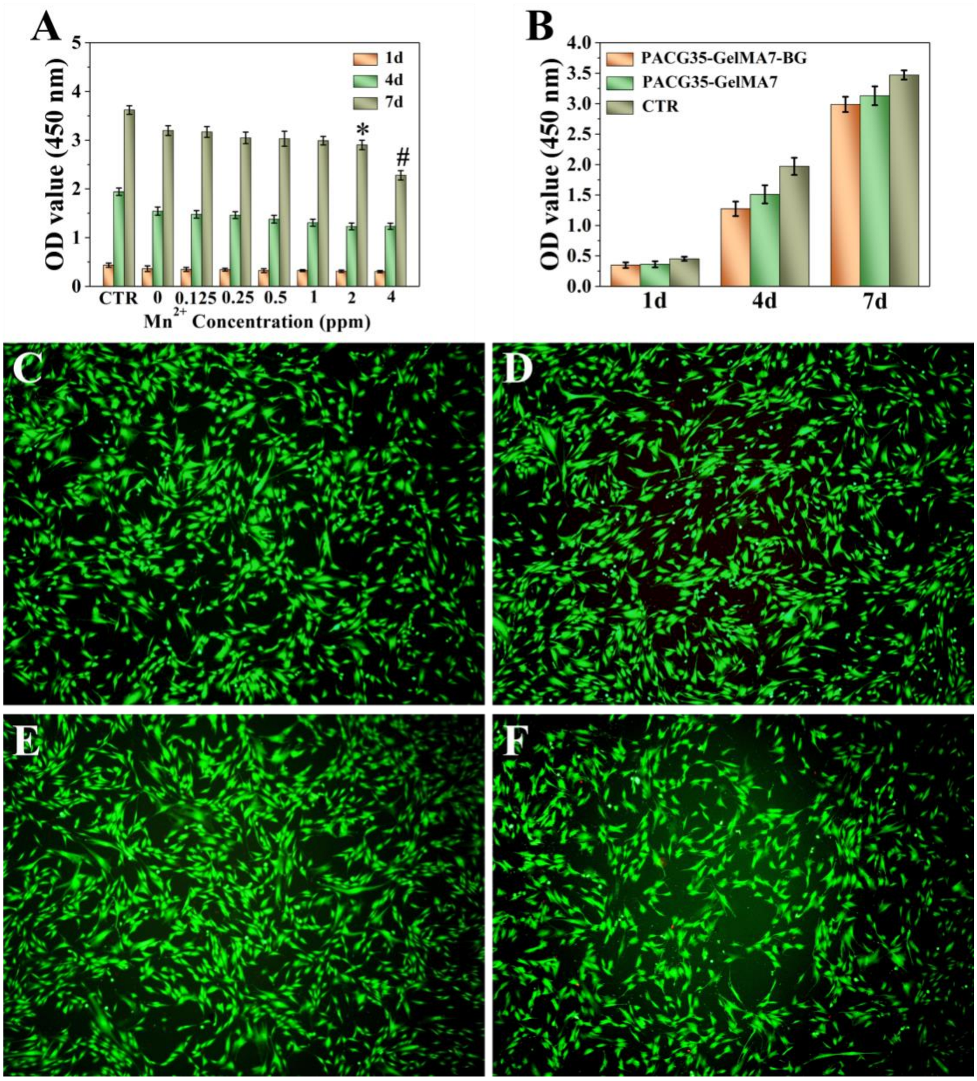

Figure S6. Proliferation of hBMSCs cultured on the surface of PACG10-GelMA10 hydrogels with different concentrations of Mn^{2+} (0 - 4 ppm) (A); The proliferation of hBMSCs cultured on the surface of PACG35-GelMA7 and PACG35-GelMA7-BG hydrogels (B); Fluorescence micrographs of hBMSCs cells cultured on the surface of the PACG10-GelMA10 (C), PACG10-GelMA10-Mn^{2+} hydrogels (D), PACG35-GelMA7 (E) and PACG35-GelMA7-BG hydrogels (F) at 24 h. The green dots indicate the living cells, while the red dots indicate the dead cells. Statistically significant differences are indicated with *p < 0.05 and #p < 0.01 compared with control groups for the same time, cell culture plate serves as a control (CTR).

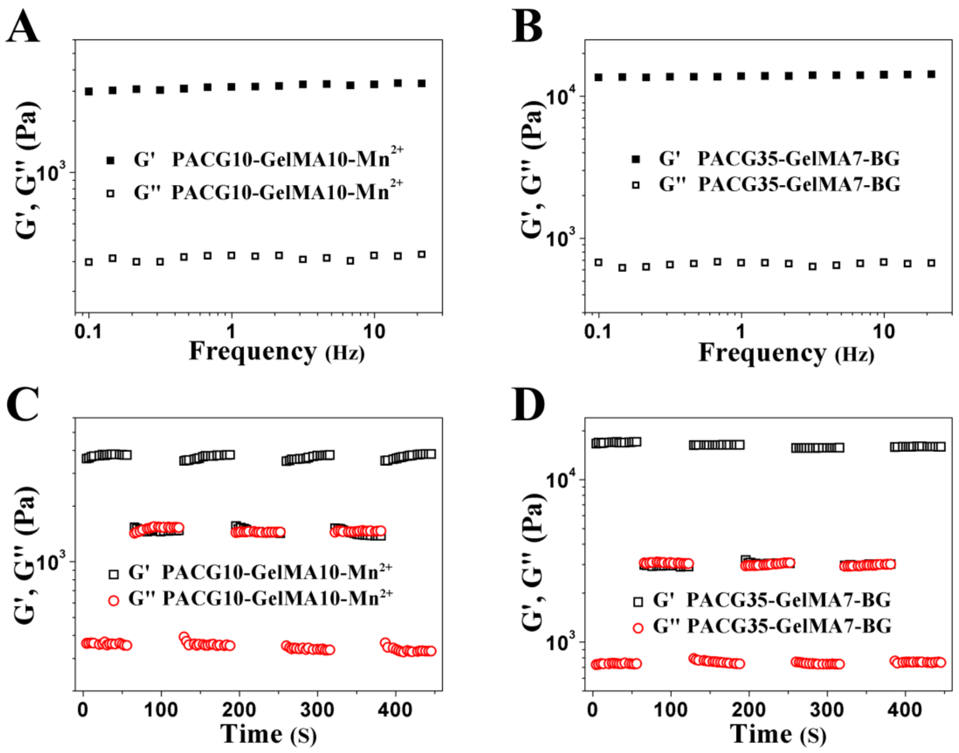

Figure S7. Rheology test of the hydrogels. Frequency sweep test of the PACG10-GelMA10-Mn^{2+} and PACG35-GelMA7-BG hydrogels (A, B); Cyclic strain sweeps with the PACG10-GelMA10 and PACG35-GelMA7-BG hydrogels alternatively being subjected to small strain (γ = 10%) and large strain (γ = 100%/300%) (C, D).

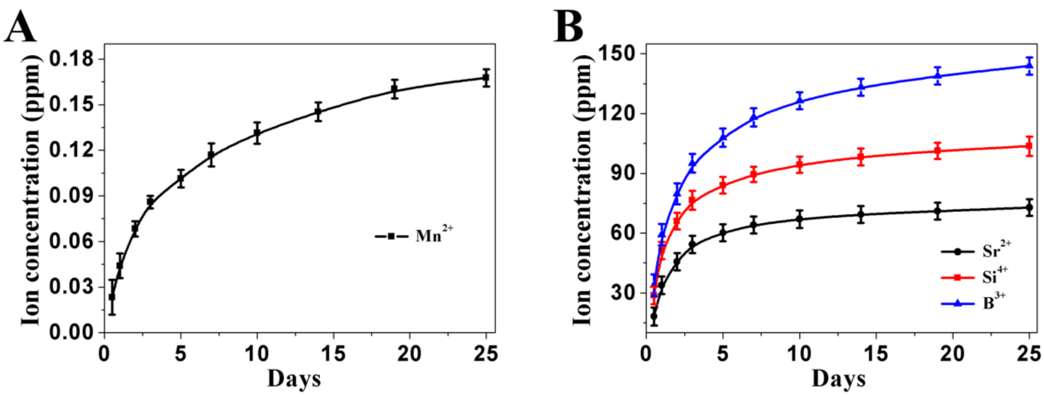

Figure S8. Accumulative release of manganese ions from PACG10-GelMA10-Mn^{2+} hydrogel scaffolds (A), and of strontium ions, silicon ions and boron ions from PACG35-GelMA7-BG hydrogel scaffolds (B) as a function of time.

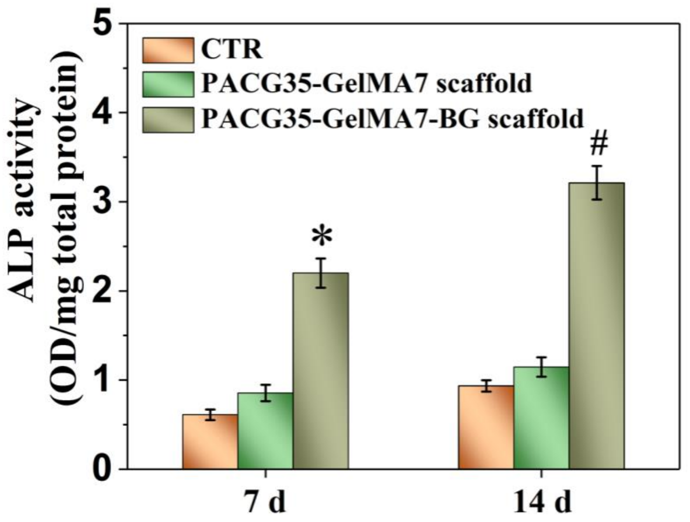

Figure S9. ALP activity analysis of hBMSCs cultured on the PACG35-GelMA7 and PACG35-GelMA7-BG hydrogel scaffold in non-osteogenic differentiation medium at days 7 and 14. Cell culture plate serves as a control (CTR). Statistically significant differences are indicated with $^{*}p < 0.05$ and $^{\#}p < 0.01$ compared with control groups for the same time.

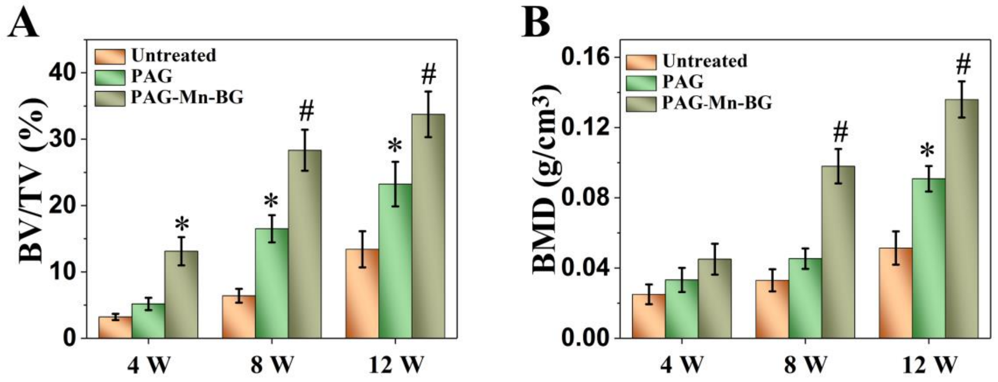

Figure S10. Quantitative micro-CT analysis of (A) the ratio of bone volume to tissue volume (BV/TV) and bone mineral density (BMD) within the subchondral bone defect region at 4, 8, 12 weeks for the three groups. Statistically significant differences are indicated with $^{*}p < 0.05$, $^{\#}p < 0.01$, compared with control group at the same time. The untreated defect (blank) serves as a control group.

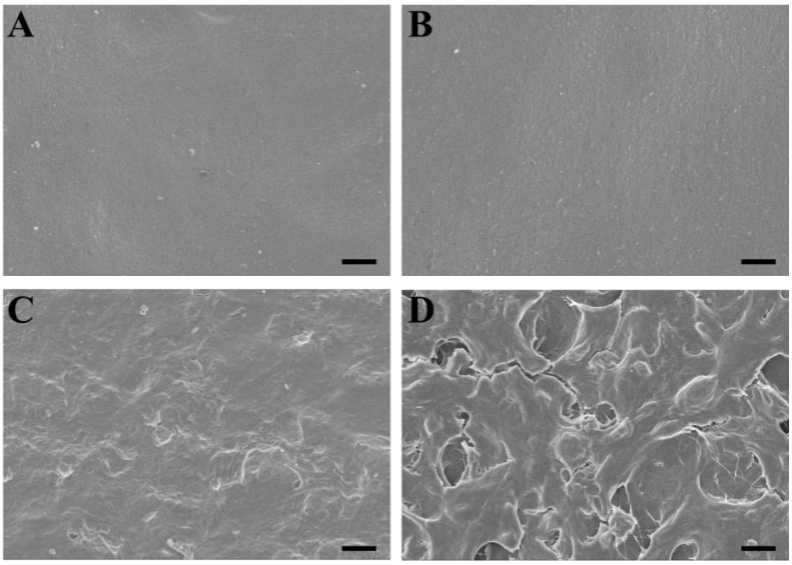

Figure S11. SEM images of the normal cartilage surface (A) and repaired cartilage surfaces at 12 weeks in PAG-Mn-BG scaffold group (B), PAG scaffold group (C), and untreated defect group (D) (Scale bar = 5 μm).

Table S1. Sequences of primers used in the real-time PCR.

Gene	Forward primer (5'-3')	Reverse primer (5'-3')
β-actin	CATGTACGTTGCTATCCAGGC	CTCCTTAATGTCCGCACGAT
AGG	ACAGCTGGGGACATTAGTGG	GTGGAATGCAGAGGTGGTTT
COL II	CAACACTGCCAACGTCCAGAT	TCTTGCAGTGGTAGGTGATGTTCT
SOX-9	CTTCCGCGACGTGGACAT	GTTGGGCGGCAGGTACTG
COL I	GGCTCCTGCTCCTCTTAG	CAGTTCTTGGTCTCGTCAC
ALP	GACCTCCTCGGAAGACACTC	TGAAGGGCTTCTTGTCTGTG
OCN	GGTGCAGACCTAGCAGACACCA	AGGTAGCGCCGGAGTCTATTCA
RUNX2	TTACCTACACCCCGCCAGTC	TGCTGGTCTGGAAGGGTCC

References:

[1] X. Cui, W. H. Huang, Y. D. Zhang, C. C. Huang, Z. X. Yu, L. Wang, W. L. Liu, T. Wang, J. Zhou, H. Wang, N. Zhou, D. P. Wang, H. B. Pan, M. N. Rahaman, *Mater. Sci. Eng. C Mater. Biol. Appl.* **2017**, *73*, 585.

[2] F. Gao, Y. Y. Zhang, Y. M. Li, B. Xu, Z. Q. Cao, W. G. Liu, *ACS Appl. Mater. Interfaces* **2016**, *8*, 8956.

[3] T. L. Wu, Z. Y. Xu, Y. Y. Zhang, H. B. Wang, C. Y. Cui, B. Chang, X. Q. Feng, W. G. Liu, *Macromol. Mater. Eng.* **2018**, *303*, 1800290.

[4] E. Hoch, C. Schuh, T. Hirth, G. E. M. Tovar, K. Borchers, *J. Mater. Sci. Mater. Med.* **2012**, *23*, 2607.

[5] Y. Liu, J. Yang, P. Zhang, C. Liu, W. Wang, W. G. Liu, *J. Mater. Chem. B* **2012**, *22*, 512.

[6] G. Eke, N. Mangir, N. Hasirci, S. MacNeil, V. Hasirci, *Biomaterials* **2017**, *129*, 188.

[7] T. W. Xin, Y. Gu, R. Y. Cheng, J. C. Tang, Z. Y. Sun, W. G. Cui, L. Chen, *ACS Appl. Mater. Interfaces* **2017**, *9*, 41168.
